# The Italian general psychiatry and forensic psychiatry treatment model: a unique story

**DOI:** 10.1017/S1092852924000646

**Published:** 2025-01-17

**Authors:** Felice F. Carabellese, Lia Parente

**Affiliations:** 1 Faculty of Medicine and Psychology, University of Rome “Sapienza”, Rome, Italy; 2 Faculty of Medicine and Psychology, Universita degli Studi di Roma La Sapienza, Rome, Italy; 3 Section of Criminology and Forensic Psychiatry, University of Bari, Bari, Italy

**Keywords:** Mental healthcare system, psychiatry hospital, high-security hospital, forensic psychiatry treatment, REMS

## Abstract

The history of Italian general psychiatry and forensic psychiatry over the last 50 years has been unique in the European and Western healthcare landscape. Western politicians often visit Italy to observe the successful community-based systems that have developed in that country.

This article represents a first step toward a necessary attempt, to explore how specific political decisions, such as the Italian one, have produced positive outcomes for patients with psychotic disorders, outcomes not observed in many Western countries, which are instead grappling with negative outcomes such as the complicated management of homelessness and the incarceration of people who would instead require psychiatric care.

In its historical context, the 1978 decision to abandon the asylum tradition in favor of socialization for patients living with severe mental disorders represented a difficult choice. This choice led to inevitable critical issues, which today are still not completely dormant.

This choice has also, undoubtedly, restored dignity to people living with serious mental illness, even when that person commits a crime.

To understand these changes, it is appropriate to mention the regulations that finally led to Law number 180 of 1978, which decreed the closure of psychiatric hospitals (Ospedale Psichiatrico) throughout Italy and continued after 2015 with the closure of high-security psychiatric hospitals (Ospedale Psichiatrico Giudiziario) as well.

Culturally, much has changed throughout this time in assistance to the mentally ill in Europe.

## Introduction

Law number 180 is the first and only framework law that imposed the closure of mental hospitals in Italy and regulated compulsory healthcare treatment, establishing public mental health services. This law made Italy the first country in the world to have abolished psychiatric hospitals.

Law 180 of 1978 is known as the Basaglia law, from the name of the psychiatrist and director of Ospedale Psichiatrico (OP) of Gorizia. Dr. Basaglia underlined the pathogenic effect of long hospitalizations in OP and suggested a different approach to severe mental illness, oriented toward community psychiatry and the rehabilitation of the patient to be implemented in his or her context of origin.

Law 180 became then part of the subsequent Law number 833 of 1978, which established a unique public National Health Care System (Sistema Sanitario Nazionale [SSN]), which in Italy guarantees free and high-quality healthcare to all citizens for hospital admissions, emergency treatments, services of general practitioners, and pediatrics choice. The SSN represents the center of health care in Italy and it is also fundamental to meet the health protection requirements provided by the Italian Constitution^1^.

The Law number 180 and subsequent Law number 833 substantially provided freedom from compulsory care to patients with mental illness, following international bioethical guidelines that identified the inviolable rights of patients to autonomy and self-determination^1^. Even a patient with severe schizophrenia spectrum disorder could usually accept or refuse the treatments, just like any other patient, according to the Italian Constitutional rights^2^.

The Law number 833 of 1978, in fact, guaranteed to psychiatric patients, freedom and right to give or deny consent to treatment. This freedom conferred on the patient eliminated any presumption according to which the state of “suffering from a mental disorder” automatically entailed the inability to give consent to treatment. In essence, the right to self-determination was returned to the psychiatric patient.

The Law number 883 radically changed mental healthcare in Italy, providing for the transition to a small open community treatment model entrusted to the public psychiatric services, instead of an asylum model. During this transition period, thousands of patients were discharged from Italian OPs^3^.

The law was followed by ~20 years of transition. “During this transition time, thousands of patients were discharged from the OPs and step by step they returned home to their families whenever possible, otherwise they were transferred to smaller residential therapeutic communities. At the same time, a new public therapeutic model was established to support the reintegration of mentally ill patients back into their family and society”.^2^ After this transition period, OPs became completely inactive due to cultural, scientific, and maybe economic resistances.

Little by little, they went back home to their families whenever possible, otherwise they were transferred to smaller general psychiatric facilities, based on the community model. These are small communities situated in the urban context with a variable number of patients (from three to 15, mostly 10), who live together and take care of all daily chores. These patients, depending on their level of functional autonomy, are assisted by a variable number of healthcare professionals. If needed, this care is provided throughout the day and at night (for those patients with a lower level of functional autonomy). For others, the care is only for a few hours a day.

Patients placed in communities gradually acquire as greater levels of functional autonomy as possible and in the most favorable cases live in small groups of two or three patients, provide for all their daily needs independently, assisted for a few hours a day by caregivers, and are included in protected work activities. In the less favorable cases, they remain in a community of 10 to 15 patients assisted throughout the day by health professionals, free to carry out rehabilitation, leisure and cultural activities, to see their family members, and to freely frequent the places of interest to them.^4^

During this time, a new public therapeutic model was established to support the reintegration of patients with severe mental illness back into their families and original social context.

From that period onwards, Italian psychiatrists refined their therapeutic rehabilitation practices for patients and family support, developing an increasingly widespread outpatient care system in the local area. These outpatient care systems are multidisciplinary, freely accessible, and focused on the patient’s needs and their level of functionality.

The relationship that the psychiatrist and other health professionals build with the patient day after day has become the true therapeutic lever of the treatment, intending to restore personal dignity. Voluntary treatment is the respected priority. Therapeutic relationships are aimed at collaboratively obtaining from the patient consent to the treatment.

Therefore, patients with severe mental disorders, including schizophrenia spectrum disorders, have attained greater economic autonomy, greater housing autonomy, welfare, and material support for their family. Additionally, patients have easy access to outpatient facilities that are open throughout the day, every day, to guarantee continuity of therapy and assistance.

Open services have been created, where patients socialize with each other and carry out a series of creative and playful activities. Policies now protect the patients, take care of their economic and personal interests, and support them in their choices.

Much has been done since then and much remains to be done, of course.

## From Asylum Treatment to Community Treatment

Before 1978, the threshold for involuntary treatment was based on the dangerousness standard, similar to standards still in practice in other Western countries today.

Royal Law no. 36 of 1904 allowed the hospitalization of mentally ill subjects into an OP indefinitely, when the patient was declared “dangerous to themselves or others” by any doctor or by a police officer. The dangerousness determination and consequent hospitalization in OP was a police measure, not a health service practice.

Once involuntarily hospitalized in an OP, the OP’s Director automatically became the patient’s legal guardian and had the sole legal power to decide if and when to discharge the patient.

Therefore, upon admission to the OP, the patient lost all personal rights for an indefinite period: the patient could not make a will, could not receive an inheritance, could not vote, and could not marry. If the patient had sexual intercourse with another person, the latter automatically was considered guilty as a sexual offender by Italian penal code. The patient essentially had no right to his own sexual life.

The subsequent Law number 431 of March 14, 1968, introduced partial modifications to the previous policy, to improve patient rights and protected autonomy; however, it was Law number 833 that restored the will of self-determination and the competence to consent to treatment for mentally ill patients as for all other citizens.

When examining how policy impacts outcomes, an important aspect of the modifications to Italian law in 1978 is the change in the criteria for determining involuntary treatment.

The involuntary hospitalization procedure was changed in fact by Law number 833 from a dangerousness standard (like that currently in use in western countries such as the United States) to a medical standard. A police measure was previously used to order mentally ill individuals considered to be “dangerous to themselves or others” to indefinite hospitalization in an OP.

The Italian regulation for civil commitment no longer considered danger to oneself or others as a prerequisite, going beyond the Royal Decree 36 of 14 February 1904, which allowed the admission to OPs of those said to be of “sick mind” and dangerous to themselves or others was changed by Law 833 of 1978. Under this new law, involuntary hospitalization became an exclusively health care act authorized by a magistrate and it could only be ordered when all of the following three conditions are met and recognized by at least two medical doctors: the need foremergency care, a treatment that required hospitalization, and incompetence to give valid consent to medical treatment due to the patient’s severe illness. This aligned the standard for involuntary treatment in schizophrenia with that for other medical conditions, away from a need for dangerousness and toward an evaluation of medical necessity and medical competence to give consent to treatment.

The involuntary hospitalization has a default duration (maximum 7 days, renewable once or twice for 7 more days at a time, subject to the approval of the same magistrate and at the request of the psychiatrist for valid medical reasons). During the entire duration of the involuntary hospitalization, the hospitalized patient is protected and safeguarded in all rights and prerogatives by a special magistrate. In both cases (voluntary and involuntary), hospitalization can occur only in a general hospital, not in OP, with newly created psychiatry wards in general hospitals consisting of small treatment units with up to 16 beds.

The transition to a medical necessity model for involuntary hospitalization, rather than a dangerousness model, has not resulted in abuses. For example, in Italy in 2021, only 7.6% of all hospital admissions were involuntary. The mean duration of hospitalizations was 12.8 days. Approximately 30% of patients admitted at discharge had a diagnosis of schizophrenic disorder. However, more than 70% of patients who receive three or more lifetime involuntary hospitalizations have schizophrenia spectrum disorders (Ministry of Health source). This is a problem of adherence to treatment which, as is known, makes the course of schizophrenic disorders problematic.

The policy change was also followed by a profound change of the psychiatrist’s professional roles and responsibilities. Initially, their tasks mainly involved patient monitoring. After the Law number 833 of 1978, their therapeutic activity became more focused on the support and strengthening of the patient’s functional autonomy and social reintegration. In this manner, Italian psychiatrists act in accordance with the fundamental constitutional rights, which were fully restored for the mentally ill patients too by the new law^4^. It revealed that Italian psychiatrists strongly rejected the previous role of social control required by the dangerousness standard.

The refusal of Italian psychiatrists to be agents of social control was evident in reform, which decreed the closure of Italian High Security Hospitals (Ospedale Psichiatrico Giudiziario [OPG]) in 2015.

Before that, on April 1, 2008, the Decree of the Italian Prime Minister implemented the prior Decree-Law (Number 230)^5^ on June 22, 1999, which transferred the responsibility of treating OPG’s patients and the mentally ill prisoners from the Department of Justice to the Department of Health. By June 30, 2010, there were 1457 men and 95 women who were socially dangerous inpatients in the six Italian OPGs.

The following Decree Law issued on December 20, 2011, number 211, provided for the transfer of the OPGs’ patients to the community facilities located in each of Italy’s 20 regions, named Residence for Execution of Security Measure (REMS), pending the closure of the OPGs initially expected by February 1, 2013.

REMS is mental health community facility under Mental Health Department (DSM) coordination each with 20 beds, suitable for the accommodation and treatment of socially “dangerous” offenders found Not Guilty by Reason of Insanity (NGRI) with a higher level of social dangerousness. REMS resemble small security hospitals, locked, secure facilities that provide inpatient treatment.

The Decree Law of March 25, 2013, postponed the closure of the OPGs until April 1, 2014. The Decree Law of March 31, 2014 (Number 52), which was modified and came into effect on May 30, 2014 (Number 81), set the final closing date of the OPGs and the discharge of all inpatients as April 1, 2015. With the law of May 30, 2014, Number 81, Italy passed from a forensic psychiatric model based on OPGs to one based on REMS in each region, from an asylum model of forensic treatment to a rehabilitative model of care. As a result, the discrepancy between general psychiatry treatment and forensic psychiatry treatment, created almost 40 years ago by Law 833 of 1978 was resolved. This means that also the “dangerous” offenders found NGRI would be treated by the same community treatment model entrusted to the public DSM for general psychiatric public services.

REMS are mental health community facilities with 20 beds operated broadly to the standard of “medium security” in other European states. Commitment to a REMS facility is designed as a custodial security measure, which is “extreme and exceptional.” Precisely because of the exceptional nature of the security measure in REMS, the total number of beds in the 32 Italian REMS is ~600 beds. In fact, the average length of stay in REMS is just over a year.^5^ The use of security measures with a lower level of security such as “*libertá vigilata*,” a sort of probation measure or supervised outpatient treatment, is more frequent. Even patients with schizophrenic disorders are more often on probation than interned in REMS and demonstrate good functional adaptation to the current community treatment and rehabilitation model. The use of rehabilitation, psychotherapy, and family support activities are common practices in the forensic treatment model of our country and allow for continuous and effective management even of patients with severe mental illnesses. A more problematic and less effective management concerns REMS patients affected by severe personality disorders, who represent approximately one-third of all REMS patients.^5^ In recent years, in fact, the need has emerged to provide for a reduced number of patients a higher level of security than that of REMS. During the validation of new assessment tools for the Italian population, this need was quantified in ~5–9% of all NGRI patients who committed crimes socially dangerous.^9^

The Law number 81 of 2014 has also raised criticism not only among psychiatrists who find themselves having to once again manage the dangerousness of their patients, a matter no longer within their competence after Law number 883 of 78, but also in the judicial world.^4^ ^,^ ^7^

In fact, in some Italian regions, especially those that make little use of recidivism risk assessment tools^3^ ^,^ ^6^ waiting lists of patients have been created before they enter REMS. How and where to manage patients during the waiting period is a problem raised above all by jurists. Before 2015, in fact, the placement of the NGRI patient in OPG occurred without any waiting, immediately after the judge’s order (Art. 222 Italian penal code) or even at the request of the prosecutor in a provisional and temporary form (Art. 206 Italian penal code). Further, this naturally allowed for a more immediate and less problematic management of the NGRI mentally ill offender at risk of criminal recidivism by the judicial authority.

The Law number 81, 2014, limits the maximum duration of internment in REMS to the maximum time of imprisonment had the offender been found guilty of the crime and was sentenced. The security measure is applied only for those NGRI patients who are considered socially dangerous, that is, at risk of criminal recidivism. The assessment of social dangerousness must be renewed periodically (every 6 months) and confirmed by the judge.

Concept of social dangerousness is still in the Italian Penal Code (Art. 203) and the judge is the only one who decides whether or not to apply security measures, REMS, or others less severe security measures. Both REMS internment and other security measures are mandatory, decided by the judge; however, the NGRI patient retains the right to consent (and refuse) to treatment^10^.

The exceptional nature of the REMS security measure derives from the intention of the Italian legislature to balance two different principles of equal dignity. On the one hand, there is the principle of patient consent, which consists in the voluntary nature of the medical treatment already recognized for non-forensic patients by Law number 833 of 1978 and now also for forensic patients who committed crimes by Law number 81 of 2014. On the other hand, there is the need to contain patients who have committed crimes with restrictive measures because of their recognized social dangerousness, because they are at a risk of criminal recidivism. By balancing the two requirements, the legislature intended to give REMS measure the character of “last resort,” but at the same time with a rehabilitative aim (Tables 1 and 2)

**Table 1.** Main Innovative Laws on Psychiatric Care in Italy

Italian Mental Health Care Laws	System Structuration	Patients’ Condition
Presence of mental asylums in Italy since the 1400s, but regulated by:		People were interned in OPs:
Royal Decree no. 36 of 1904, “Provisions on mental asylums and the mentally ill” (OP management by Italian provincial). Royal Decree no. 615 of 1909 “Regulations on mental asylums and the mentally ill” (OP management by Italian provincial).	In 1904 there were 98 OPs in Italy In all 98 OPs there were 89 000 patients	without consent for a dangerousness standard with a police measure internment for indefinite time losing all rights OP’s Director automatically became the patient’s legal guardian
Law no. 431 of March 14, 1968	creation of provincial day hospitals each OP divided into sections with 125 beds no automatic Director’s legal guardian	greater psychological and rehabilitative attention possibility of voluntary admission of the patient to OPs
Law no. 833 of 1978 (that incorporated the Law no. 180 of 1978)	establishment of the SSN closure of OPs psychiatric wards in General Hospitals community model of care mental health care as a health service practice involuntary hospitalization as an exclusively health care act authorized by a magistrate	patient’s right to self-determination for treatment patient’s right to freely consent to treatment other civil rights to patient rehabilitation goals

**Table 2.** Main Innovative Laws on Forensic Psychiatric Care in Italy

Italian Forensic Mental Health Care Laws	System Structuration	Patients’ Condition
Italian Security Hospital		
1876 Aversa is the first Italian “manicomio criminale” a second one in Montelupo Fiorentino in 1886 a third in Reggio Emilia (1892) a fourth in Naples (1922) a fifth in Barcellona Pozzo di Gozzo in 1925 finally, one in Castiglione delle Stiviere in 1939 In 1975 the “manicomi criminali” were called “Ospedali Psichiatrici Giudiziari” (OPG)	Section for “maniacs”	Internment in Security Hospital as a security measure (Art. 203 Italian Penal Code) for NGRI offenders About 200 patients for each OPG
Constitutional Court Sentence on July 18, 2003, no. 253	Admission to a different security measure (*libertà vigilata*)	1457 men and 95 women in all OPGs at 2010
Decree of the Italian Prime Minister of April 1st, 2008, implemented the prior Decree Law (Number 230) on June 22, 1999	The responsibility of treating OPG’s patients and the mentally ill prisoners was transferred from Department of Justice to Department of Health	Max 20 patient for each REMS
Decree Law December 20, 2011, no. 211	Transfer of the OPGs’ patients to the REMS in each Italian region	
Law no. 81, May 30, 2014	OPG closure	At 2023, there are 32 REMS in Italy

## Conclusion

From this review and analysis of Italy’s transformation mental health services for forensic patients requiring security measures, we cannot draw comparisons between the civil mental health systems in Italy and other countries such as the United States. Deinstitutionalization without development of adequately robust community mental health services is an often cited factor in the relentless increase in mentally ill persons entering US jails and prisons (Torrey et al, 2014). However, the Italian model does demonstrate that, with a robust community system in place, deinstitutionalization is possible without increases in criminalization.

Furthermore, Italy’s transition to an informed consent model for involuntary treatment, and away from a dangerousness model, appears to have effectively reduced the need for involuntary treatment. The change in involuntary treatment standard in the context of community treatment has also provided for improved dignity and strengthened therapeutic relationships between psychiatrists and people living with schizophrenia.^8^ Policy makers visiting Italy for seeing it’s model mental health systems should take these factors into consideration.

## Footnotes

1 Italian Constitution, Article 32, I paragraph: “the Republic protects health as a fundamental right of the individual and in the interest of the community.”

2 Italian Constitution, Article 13: “Personal freedom is inviolable” and Italian Constitution, article 32, II paragraph: “No one can be forced to a certain medical treatment if not for provision of law.”

3 In 1978, when the 98 Italian OPs closed, there were ~89 000 patients inside them.

4 Italian Constitution, Article 2: “The Republic recognizes and guarantees the inviolable human rights.” Note that the Law 833 abolished the automatic recourse to legal protection for a mentally ill subject at his entry into the OP.

5 Decree of the Prime Minister: Procedure and criteria for the transfer of health function to the National Health Care Service, work relationships, financial resources, and equipment and instruments in matters of correctional health (General Series no. 126 of May 30, 2008). This decree established and described the transfer of all treatment of mentally ill prisoners in Italy from the Department of Justice to the Department of Health.
